# An Acoustic Flat Lens for Broadband Focusing via Cross-Shape Structure

**DOI:** 10.3390/mi14010012

**Published:** 2022-12-21

**Authors:** Shenlian Gao, Qinglei Zeng, Mengyang Gong, Jun Lan, Xiaozhou Liu

**Affiliations:** 1 Key Laboratory of Modern Acoustics, Institute of Acoustics, School of Physics, Collaborative Innovation Center of Advanced Microstructures, Nanjing University, Nanjing 210093, China; 2 College of Computer Science and Technology, Nanjing Tech University, Nanjing 211800, China; 3 State Key Laboratory of Acoustics, Institute of Acoustics, Chinese Academy of Sciences, Beijing 100190, China

**Keywords:** broadband focusing, soundwave manipulation, metamaterials, microscale

## Abstract

The manipulation of refracted wavefronts is eye-catching for owning attractive applications. In this article, an airborne acoustic flat lens for broadband focusing via cross-shape structure was proposed and demonstrated, introducing the broadband manipulation of wavefronts. The designed metasurface employs gradient refractive index cells to redirect the sound wave. Based on our theory, the effective refractive indexes of our unit cells can be easily calculated. The shackle of narrowband metasurfaces is conquered, and applications in medical ultrasound imaging are just around the corner.

## 1. Introduction

Various domains show the urgent requirements of microscale metasurfaces for achieving sound fields with multiple functions, such as anomalous refraction, acoustic focusing, and acoustic bending by microscale [1,2,3,4,5,6,7,8,9]. Various sub-wavelength thickness planar lenses have been designed. Some of the metasurfaces can be considered as pipes that change the length of the sound path, and some of them can be taken as resonance structures [10,11,12,13,14,15,16,17,18,19]. So, many of them are sensitive to frequency, which means the metasurface shaping wavefronts work within narrowband frequencies [20]. Former researchers designed broadband materials to regulate refracted wavefronts while their models lack valid theoretical proofs [21,22].

Our optimized metasurface for broadband focusing consists of cross-shape plates. The effective refractive indexes of our unit cells can be altered conveniently by simply changing the number of cross-shape plates. The unit cells are different in the number of cross-shape plates and are essential for the construction of flat lenses, and the gradient refractive indexes can be realized.

## 2. Theory

**Figure 1:**
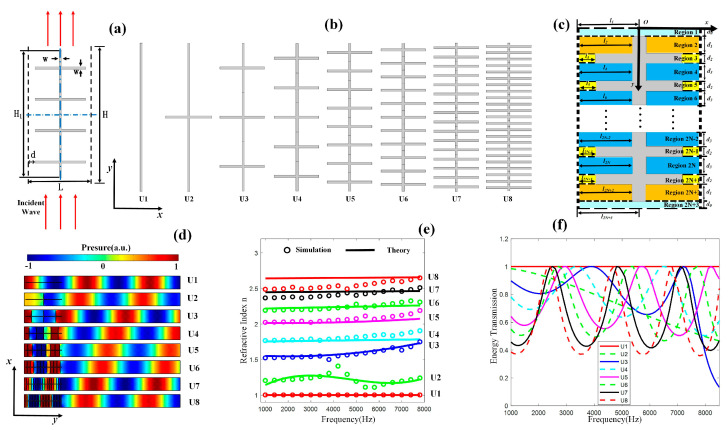
(**a**) The design of our units. (**b**) The units different in the number of cross-shape plates. (**c**) Detailed exhibition of a unit cell. (**d**) Simulated sound pressure field patterns of our units at 7100 Hz where the sound pressure is normalized to the incident plane wave. (**e**) The theoretical and numerical effective refractive indexes of our units at different frequencies. (**f**) The numerical effective energy transmission coefficient of the units.

Our unit cells consist of linked cross-shape plates. Figure 1a shows the design of our unit cells. *H*, *H*_1_, *L*, *W*, and *d* represent the length of our unit cells, the total length of cross-shape plates, the width of unit cells, the width of the cross-shape plates, and the width of the air channel, respectively. We fixed *H* = 30 mm, *H*_1_ = 28 mm, *L* = 10 mm, *W* = 0.2 mm, and *d* = 0.5 mm.

*W* is optimized for higher transmissivity and better stability. If *W* is too wide, the cross-shaped plates may block the sound path, and the transmissivity of our unit cells will drop. If *W* is too narrow, the unit cells may not work properly for changing the phase of the soundwave at low frequencies, and the stability will be broken at high frequencies. The distance between the centers of two cross-shape plates is 28/*N* mm. *N* represents the number of the cross-shape plates. When the soundwave propagates through the cross-shape plates, the phases of the soundwave can be delayed. The phase of the output soundwave in the air can be modified by changing the number of cross-shape plates. The numbers of the cross-shape plates of the units are selected as 0, 1, 3, 5, 8, 11, 16, and 23, respectively. The design of the units is shown in Figure 1b. 

The detailed exhibition of our units is shown in Figure 1c. The unit cell with *N* cross-shape plates can be divided into 2*N* + 3 regions. *d*_0_ is 1 mm, *d*_1_ is 0.5*(*H*_1_ − N*W)/*N* mm, *d*_2_ is 0.2 mm, and *d*_3_ is (*H*_1_ − N*W)/*N* mm. The y coordinate of the boundaries of the adjacent regions is *y_m_* (m = 1, 2, …, 2*N* + 2, *y*_1_ = *d*_0_). The sound pressure at different regions is shown as follows:

(1) $$\begin{array}{l} \left\{\begin{array}{l} p_{i}^{\left(1\right)}=e^{-jky}\\ p_{r}^{\left(1\right)}=r_{p}e^{jky} \end{array}\right.\\ \left\{\begin{array}{l} p_{i}^{\left(m\right)}=A_{m}e^{-jk(y-y_{m-1})}\\ p_{r}^{\left(m\right)}=B_{m}e^{jk(y-y_{m-1})} \end{array}\right.\\ p_{t}=t_{p}e^{-jk(y-y_{2N+2})} \end{array}$$

where $p_{i}^{\left(m\right)}$ represents the transmitted sound pressure and $p_{r}^{\left(m\right)}$ represents the reflected sound pressure in the m^th^ region. *p_t_* represents the transmitted sound pressure of our unit cell. *A_m_* and *B_m_* represent the coefficients of the sound pressure in the m^th^ region, respectively. *l_m_* represents the width of the m^th^ region. *t_p_* and *r_p_*, respectively, represent the sound pressure transmission coefficient and reflection coefficient. *k* represents the wave number of the incident sound wave.

Based on the acoustic continuity conditions (the continuities of sound pressure and volume velocity) on the boundaries of the connected regions, the matrixes at different positions inside the structure can be deduced to relate sound pressure and volume velocity. The equations can be shown as follows:

(2) $$\begin{array}{l} y=y_{1}:\left\{\begin{array}{l} 1+r_{p}=A_{2}+B_{2}\\ l_{1}(1-r_{p})=l_{2}(A_{2}-B_{2}) \end{array}\right.\\ y=y_{m}:\left\{\begin{array}{l} A_{m}e^{-jk(y_{m}-y_{m-1})}+B_{m}e^{jk(y_{m}-y_{m-1})}=A_{m+1}+B_{m+1}\\ l_{m}(A_{m}e^{-jk(y_{m}-y_{m-1})}-B_{m}e^{jk(y_{m}-y_{m-1})})=l_{m+1}(A_{m+1}-B_{m+1}) \end{array}\right.,(m=2,3,\ldots ,2N+1)\\ y=y_{2N+2}:\left\{\begin{array}{l} A_{2N+2}e^{-jk(y_{2N+2}-y_{2N+1})}+B_{2N+2}e^{jk(y_{2N+2}-y_{2N+1})}=t_{p}\\ l_{2N+2}(A_{2N+2}e^{-jk(y_{2N+2}-y_{2N+1})}-B_{2N+2}e^{jk(y_{2N+2}-y_{2N+1})})=l_{2N+3}t_{p}. \end{array}\right. \end{array}$$

Matrices are adapted to denote the boundary equations and the matrixes are shown as follows:

(3) $$\begin{array}{l} y=y_{1}:\left(\begin{array}{cc} 1 & 1\\ 1 & -1 \end{array}\right)\left(\begin{array}{l} 1\\ r_{p} \end{array}\right)=\left(\begin{array}{cc} 1 & 0\\ 0 & \frac{l_{2}}{l_{1}} \end{array}\right)\left(\begin{array}{cc} 1 & 1\\ 1 & -1 \end{array}\right)\left(\begin{array}{l} A_{2}\\ B_{2} \end{array}\right)\\ y=y_{m}:\left(\begin{array}{cc} 1 & 1\\ 1 & -1 \end{array}\right)\left(\begin{array}{cc} e^{-jk(y_{m}-y_{m-1})} & 0\\ 0 & e^{jk(y_{m}-y_{m-1})} \end{array}\right)\left(\begin{array}{l} A_{m}\\ B_{m} \end{array}\right)=\left(\begin{array}{cc} 1 & 0\\ 0 & \frac{l_{m+1}}{l_{m}} \end{array}\right)\left(\begin{array}{cc} 1 & 1\\ 1 & -1 \end{array}\right)\left(\begin{array}{l} A_{m+1}\\ B_{m+1} \end{array}\right),(m=2,3,\ldots ,2N+1)\\ y=y_{2N+2}:\left(\begin{array}{cc} 1 & 1\\ 1 & -1 \end{array}\right)\left(\begin{array}{cc} e^{-jk(y_{2N+2}-y_{2N+1})} & 0\\ 0 & e^{jk(y_{2N+2}-y_{2N+1})} \end{array}\right)\left(\begin{array}{l} A_{n}\\ B_{n} \end{array}\right)=\left(\begin{array}{cc} 1 & 0\\ 0 & \frac{l_{2N+3}}{l_{2N+2}} \end{array}\right)\left(\begin{array}{cc} 1 & 1\\ 1 & -1 \end{array}\right)\left(\begin{array}{l} t_{p}\\ 0 \end{array}\right). \end{array}$$

Equation (3) can be further simplified as follows:

(4) $$\begin{array}{l} \left(\begin{array}{l} 1\\ r_{p} \end{array}\right)={\left(\begin{array}{cc} 1 & 1\\ 1 & -1 \end{array}\right)}^{-1}\left(\begin{array}{cc} 1 & 0\\ 0 & \frac{l_{2}}{l_{1}} \end{array}\right)\left(\begin{array}{cc} 1 & 1\\ 1 & -1 \end{array}\right)\left(\begin{array}{l} A_{2}\\ B_{2} \end{array}\right)\\ \left(\begin{array}{l} A_{m}\\ B_{m} \end{array}\right)=\left(\begin{array}{cc} e^{jk(y_{m}-y_{m-1})} & 0\\ 0 & e^{-jk(y_{m}-y_{m-1})} \end{array}\right){\left(\begin{array}{cc} 1 & 1\\ 1 & -1 \end{array}\right)}^{-1}\left(\begin{array}{cc} 1 & 0\\ 0 & \frac{l_{m+1}}{l_{m}} \end{array}\right)\left(\begin{array}{cc} 1 & 1\\ 1 & -1 \end{array}\right)\left(\begin{array}{l} A_{m+1}\\ B_{m+1} \end{array}\right),(m=2,3,\ldots ,2N+1)\\ \left(\begin{array}{l} A_{2N+2}\\ B_{2N+2} \end{array}\right)=\left(\begin{array}{cc} e^{jk(y_{2N+2}-y_{2N+1})} & 0\\ 0 & e^{-jk(y_{2N+2}-y_{2N+1})} \end{array}\right){\left(\begin{array}{cc} 1 & 1\\ 1 & -1 \end{array}\right)}^{-1}\left(\begin{array}{cc} 1 & 0\\ 0 & \frac{l_{2N+3}}{l_{2N+2}} \end{array}\right)\left(\begin{array}{cc} 1 & 1\\ 1 & -1 \end{array}\right)\left(\begin{array}{l} t_{p}\\ 0 \end{array}\right),\\ \left(\begin{array}{l} 1\\ r_{p} \end{array}\right)={\left(\begin{array}{cc} 1 & 1\\ 1 & -1 \end{array}\right)}^{-1}\left(\begin{array}{cc} 1 & 0\\ 0 & \frac{l_{2}}{l_{1}} \end{array}\right)\left(\begin{array}{cc} 1 & 1\\ 1 & -1 \end{array}\right)\left(\begin{array}{l} A_{2}\\ B_{2} \end{array}\right)\\ ={\left(\begin{array}{cc} 1 & 1\\ 1 & -1 \end{array}\right)}^{-1}\left(\begin{array}{cc} 1 & 0\\ 0 & \frac{l_{2}}{l_{1}} \end{array}\right)\left(\begin{array}{cc} 1 & 1\\ 1 & -1 \end{array}\right)\left(\begin{array}{cc} e^{jk(y_{2}-y_{1})} & 0\\ 0 & e^{-jk(y_{2}-y_{1})} \end{array}\right){\left(\begin{array}{cc} 1 & 1\\ 1 & -1 \end{array}\right)}^{-1}\left(\begin{array}{cc} 1 & 0\\ 0 & \frac{l_{3}}{l_{2}} \end{array}\right)\left(\begin{array}{cc} 1 & 1\\ 1 & -1 \end{array}\right)\left(\begin{array}{l} A_{3}\\ B_{3} \end{array}\right)\\ ={\left(\begin{array}{cc} 1 & 1\\ 1 & -1 \end{array}\right)}^{-1}\left(\begin{array}{cc} 1 & 0\\ 0 & \frac{l_{2}}{l_{1}} \end{array}\right)\left(\begin{array}{cc} 1 & 1\\ 1 & -1 \end{array}\right)\left(\begin{array}{cc} e^{jk(y_{2}-y_{1})} & 0\\ 0 & e^{-jk(y_{2}-y_{1})} \end{array}\right){\left(\begin{array}{cc} 1 & 1\\ 1 & -1 \end{array}\right)}^{-1}\left(\begin{array}{cc} 1 & 0\\ 0 & \frac{l_{3}}{l_{2}} \end{array}\right)\left(\begin{array}{cc} 1 & 1\\ 1 & -1 \end{array}\right)\cdot \cdot \cdot \left(\begin{array}{l} t_{p}\\ 0 \end{array}\right). \end{array}$$

According to Equation (4), the iterative method is adapted to solve the matrices. *t_p_* and *r_p_* can be calculated accordingly. 

In fact, *d* and *d*_2_ are much smaller than the wavelength. According to J. Kergomard’s theory of length correction of the discontinuities, *d*_2_ should be modified [23]. The effective length *d*_2_^′^ after correction can be calculated by the following equation:

(5) $$d_{2}{}^{\prime }=d_{2}+0.85*d_{2}*(0.8980*\log^{3}{}_{10}(N)-2.9846*\log^{2}{}_{10}(N)+1.9496*\log_{10}(N)+2.4121)$$

According to Equations (4) and (5), *t_p_^′^* and *r_p_^′^* can be calculated, where *t_p_^′^* and *r_p_^′^* represent the sound pressure transmission coefficient and reflection coefficient after correction, respectively. Based on Vladimir Fokin’s theory, the effective refractive indexes of our unit cells can be retrieved and are expressed as [24]:

(6) $$n=\frac{\pm \cos^{-1}(\frac{1}{2t_{p}{}^{\prime }}[1-{\left(r_{p}{}^{\prime }\right)}^{2}+{\left(t_{p}{}^{\prime }\right)}^{2}])}{k*H}+\frac{2\pi a}{k*H}$$

where *a* represents the branch number of the cos^−1^ function and *n* represents the theoretical effective refractive index. The cross-shape structure can realize a high refractive index. Flat lenses can be realized by the cross-shape structure.

## 3. Numerical Calculation and Analysis

Phase deviation from 0 to 2π can be realized at 7100 Hz. The sound pressure field distributions of our unit cells are shown in Figure 1d. The sound pressure is normalized to the incident plane wave. Figure 1e shows the theoretical and numerical effective refractive indexes of the units at different frequencies. The solid lines are theoretical results, and the dots are simulated results conducted by the commercial software COMSOL (5.6, COMSOL, Inc., Stockholm, Sweden). Figure 1f shows the numerical effective energy transmission coefficient of the units at different frequencies. We set the frequency range for our research between 3000 Hz and 7700 Hz so that the thickness of the designed metasurface is around 27% to 74% of the wavelength. The transmissivity of the units is over 0.5 at most of the frequencies between 3000 Hz and 7700 Hz. 

We assume the cross-shape structure as hard boundaries in our theory. The resonance of the structure attributes to the deviations in the refractive indexes of U2 at around 4200 Hz.

The numerical refractive indexes of U4 are higher than the theoretical ones at around 7500 Hz. That is because the correction of *d*_2_ is based on the theory of the radiation of the piston at low frequencies. When the frequency is relatively high (k*d > 0.5), there may be deviations between theoretical and numerical results.

The theoretical effective refractive indexes of U8 are larger than the numerical ones at low frequencies. That is because the length between two cross-shape plates (*d*_3_) is much shorter than the wavelength and is of the same order of magnitude as our cross-shape plates’ width (*d*_2_). The effective length between the two cross-plates is longer than *d*_3_. When the number of the cross-shape plates is small, the length between the two cross-shape plates (*d*_3_) is much longer than our cross-shape plates’ width (*d*_2_). In this case, the correction of the length between the two cross-shape plates (*d*_3_) can be ignored. 

**Figure 2:**
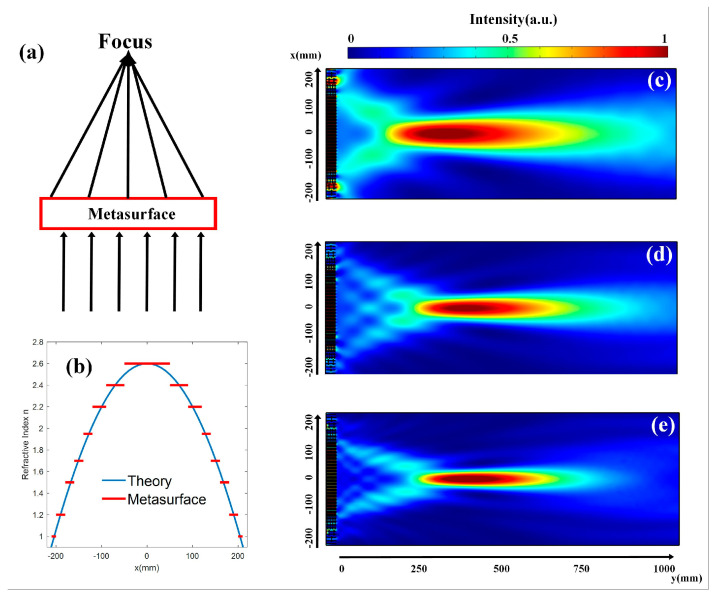
(**a**) The design of the flat lens. (**b**) The refractive index distribution of the ideal flat lens (blue lines) and that of our flat lens (red lines). (**c**–**e**) The normalized simulated sound intensity distributions of our lens at the frequencies of 3000 Hz, 5100 Hz, and 7700 Hz. The intensity is normalized to the maximum values of the sound intensity at different frequencies, respectively.

Acoustic subwavelength flat lenses for broadband focusing on the air can be realized through the cross-shape structure. Using a flat lens, we can focus the broadband plane wave to a certain position (0, *y*_0_). Figure 2a shows the schematic diagram of the lens. When the focal length $y_{0}=410\;\mathrm{mm}$, the ideal distribution of the refractive index along the x-axis is decided by the following formula:

(7) $$n(x)=-\frac{\sqrt{x^{2}+y_{0}{}^{2}}}{H}+\frac{y_{0}}{H}+n(0)$$

where $n\left(0\right)=2.61$ represents the max refractive index of the designed units. The refractive index distribution of the ideal flat lens (blue lines) and that of our flat lens (red lines) are shown in Figure 2b. Figure 2c–e shows the focusing effect of the flat lens at the frequencies of 3000 Hz, 5100 Hz, and 7700 Hz. The simulated sound intensity distributions of our flat lens are normalized to their own maximum values. From the figures, we can notice the bright focal regions with high sound intensity. The high efficiency of our flat lens can be demonstrated as the sound intensity of the input side is quite low. 

## 4. Experiment and Discussion

**Figure 3:**
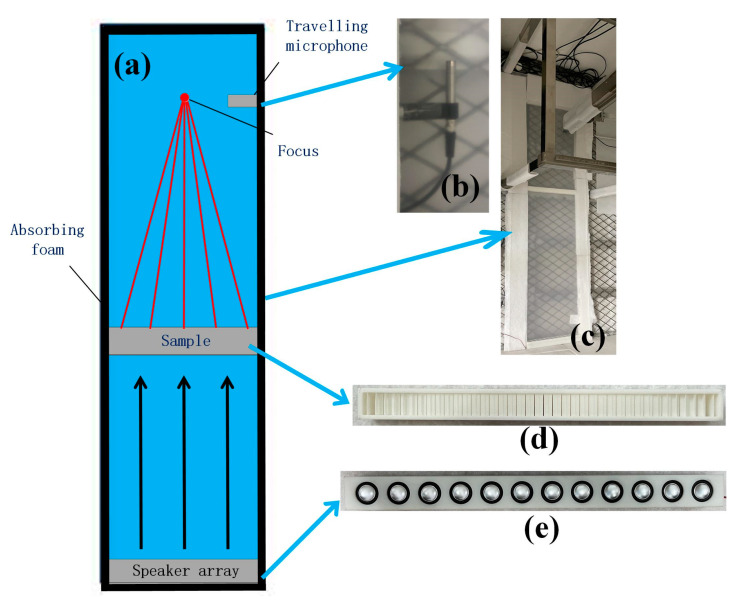
(**a**) The overall layout of the experimental equipment. Photos of (**b**) the travelling microphone, (**c**) the experimental system, (**d**) the fabricated meta-lens sample, and (**e**) the speaker array.

An experimental system is built for the further demonstration of focusing the function of our flat lens. Figure 3a–e shows the overall layout of the experimental equipment, the photos of the traveling microphone, the experimental system, the sample, and the speaker array. The flat lens is fabricated by 3D printers. The width of our metasurface is 400 mm, while the height is 35 mm. Two pieces of plastic board (1.9 m in length, 0.6 m in width, and 5 mm in thickness) are adopted to satisfy the requirement of the planar waveguide system. Absorbing foam is installed in the boundaries of the experimental platform to reduce the reflected sound of the boundaries. Here, the speaker array is used to generate incident plane waves in the waveguide. The transmission fields can be measured via a traveling microphone carried by step motors. 

**Figure 4:**
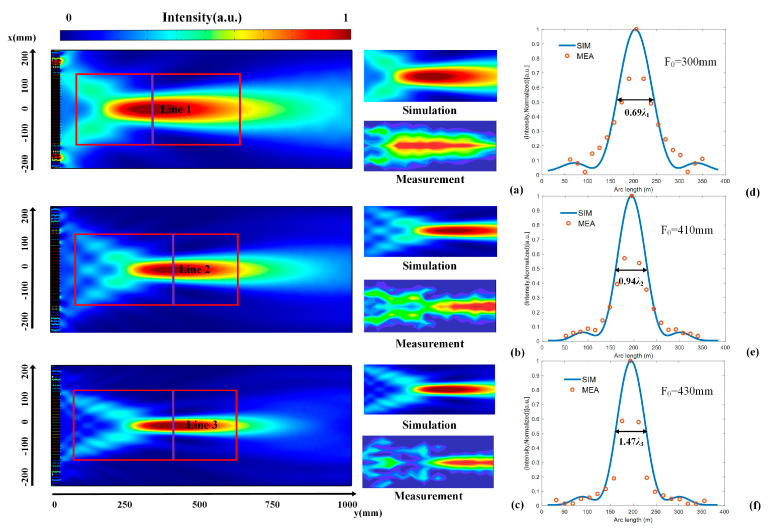
Simulated and measured focusing effect of the flat lens at (**a**) 3000 Hz, (**b**) 5100 Hz, and (**c**) 7700 Hz. Normalized simulated and measured intensity distributions on (**d**) line 1, (**e**) line 2, and (**f**) line 3. The intensity in the measured area is normalized to the maximum values of the sound intensity at different frequencies, respectively.

Figure 4a–c shows the simulated and measured focusing effect of the tested area, shown by the red rectangle area. For a given F_0_, the changes in the normalized sound intensity along the x direction, corresponding to the purple lines (lines 1–3), are shown in Figure 4d–f, respectively. Here, F_0_ is the experimental focal length of our flat lens. The broadband focusing effect of the flat lens can be observed. The simulated results (blue lines) tally with the experimental results (red dots), which confirm the broadband focusing function of our metasurface. The full width at half maximums (FWHMs) of our flat lens is 0.69 λ_1_, 0.94λ_2_, and 1.47λ_3_, respectively. λ_1_, λ_2_, and λ_3_ represent the wavelengths at the frequencies of 3000 Hz, 5100 Hz, and 7700 Hz, respectively.

Our metasurface cannot be simply taken as pipes or resonance structures. The cross-shaped structure is the fusion of pipes and resonance structures that possess the sound path with branches, and this is the key to breaking the narrowband limit. In this article, by changing the number of the cross-shape plates, we change the length of the pipes of the width of the resonance simultaneously. In fact, we can change the geometric shapes of the pipes and resonance structures, respectively, to break the narrowband limit. Compared with traditional gradient lenses, the refractive index of the fusions of pipes and resonances can be easily calculated and redesigned for other lenses. When the number of cross-shape plates changes, the effective length of the sound path changes. When the frequency changes, the cross-shape plates work, and the phase of the sound remains the same.

## 5. Conclusions

In summary, the flat lens for broadband focusing based on the cross-shape structure has been investigated. Based on the designed unit cells, broadband wavefront manipulations can be realized. The cross-shape structure provides a simple and efficient method for manipulating broadband wavefronts. By redesigning the cross-shape structure, more interesting applications can be found, and by designing new fusions of pipes and resonance, the narrowband limit can be broken.
